# Integrative transcriptomics and human tibial artery validation identify *ADAM12*, *PDK4*, and *RUNX1* as peripheral artery disease-associated genes enriched in calcified tibial arteries

**DOI:** 10.1016/j.jvssci.2026.100434

**Published:** 2026-07-27

**Authors:** Snow Adler, Jorge RuizdelRio, Brian Eliceiri, Ann C. Gaffey

**Affiliations:** a Department of Surgery, Naval Medical Center San Diego, San Diego, CA; b Department of Surgery, University of California San Diego, La Jolla, CA; c Vascular and Endovascular Surgery, University of California San Diego, La Jolla, CA

**Keywords:** Peripheral artery disease, Medial arterial calcification, Transcriptomics, ADAM12, PDK4, RUNX1

## Abstract

**Background:**

Medial arterial calcification of tibial vessels is a major obstacle to limb salvage in peripheral artery disease (PAD), yet the molecular drivers remain poorly defined. We used publicly available transcriptomic data to nominate candidate genes relevant to vascular calcification and then tested those candidates in human tibial arteries and in primary PAD-derived vascular cells.

**Methods:**

Differential gene expression was analyzed across publicly available PAD/chronic limb-threatening ischemia (CLTI) transcriptomic datasets, comparing PAD/CLTI tissue to non-PAD controls (limma and DESeq^2^ adjusted *P* < .05, |log_2_ fold-change| > 1.5). Candidates were prioritized based on predicted roles in calcification processes in endothelial cells and vascular smooth muscle cells. Expression was validated by quantitative polymerase chain reaction in isolated human tibial arteries harvested from patients with PAD/CLTI undergoing below-the-knee amputations and non-PAD control vessels. Following the preparation of primary PAD-derived endothelial cells and vascular smooth muscle cells, the cells were cultured under high-glucose or osmotic control conditions for 7 days to assess the effects of a hyperglycemic challenge.

**Results:**

Analysis of 14 datasets identified 89 differentially expressed genes enriched in inflammatory, osteogenic, and metabolic pathways. Fourteen candidates were selected for validation. Among them, *ADAM12*, *PDK4*, and *RUNX1* were confirmed in PAD tibial arteries as the most relevant candidates from the prespecified panel, consistent with their prioritization from the transcriptomic and literature-guided candidate-selection strategy.

**Conclusions:**

A candidate-first translational strategy integrating public transcriptomic prioritization with validation in human tibial arteries confirmed *PDK4* and *RUNX1* as significantly upregulated medial arterial calcification-associated genes in PAD tibial arteries, and identified *ADAM12* as a biologically prioritized candidate showing a concordant but statistically nonsignificant trend in the same direction. Their expression in diseased tissue, together with the absence of acute glucose responsiveness in primary PAD-derived vascular cells, supports a model in which these genes are linked to chronic vascular remodeling rather than transient hyperglycemic signaling. These findings refine the molecular landscape of PAD-associated calcification and establish a focused framework for potential mechanistic studies.

**Clinical relevance:**

Tibial medial arterial calcification is a major barrier to limb salvage in patients with peripheral artery disease (PAD)/chronic limb-threatening ischemia because it makes revascularization more difficult and less durable. By validating candidate genes in human tibial arteries and primary PAD-derived vascular cells, this study links diseased tissue biology to pathways that may help explain why some patients develop severe calcification and experience poor clinical outcomes. These findings provide a disease-specific framework for identifying potential biomarkers and therapeutic targets to improve infrapopliteal revascularization outcomes and reduce limb loss.

**Article Highlights**

• **Type of Research:** Human study
• **Key Findings:** We used a candidate-first strategy to move from literature-guided transcriptomic prioritization to tibial artery validation, nominating *ADAM12*, *PDK4*, and *RUNX1* as peripheral artery disease (PAD)-associated genes enriched in calcified vessels. *PDK4* and *RUNX1* were significantly upregulated in PAD, *ADAM12* showed a concordant PAD-restricted trend, and acute 7-day high-glucose exposure in PAD-derived endothelial cells and vascular smooth muscle cells did not further induce these genes, implicating chronic PAD-associated remodeling rather than short-term hyperglycemia as a key influence on their expression.
• **Take Home Message:** *ADAM12*, *PDK4*, and *RUNX1* emerged as PAD-associated genes enriched in calcified tibial arteries; however, they have not yet been established as drivers of medial calcification. Their expression pattern, together with a lack of acute glucose responsiveness in PAD-derived endothelial cells and vascular smooth muscle cells, points toward chronic PAD-associated vascular remodeling rather than short-term hyperglycemia as the dominant context in which these pathways are engaged.

Peripheral artery disease (PAD) affects hundreds of millions of adults worldwide and remains a leading cause of lower-extremity amputation.^1^^,^^2^ Chronic limb-threatening ischemia (CLTI), the most advanced form of PAD, is associated with high rates of limb loss and mortality despite contemporary revascularization strategies.^3^^,^^4^ Durable limb salvage depends on effective restoration of perfusion to the foot, most commonly through infrapopliteal endovascular or surgical interventions.

Medial arterial calcification (MAC) of tibial arteries is a major barrier to successful revascularization.^5^ Heavily calcified tibial vessels are difficult to treat with standard angioplasty, even when vessel preparation techniques are used, and they also pose substantial challenges for open surgical bypass. Severe calcification limits vessel expansion, impairs device passage, and reduces procedural success, contributing to higher restenosis and reocclusion rates.^6^ Despite its clinical significance, no therapies directly target tibial MAC.^7^ Current PAD medical treatments, including antiplatelet agents, statins, and risk-factor modification, have minimal impact on established MAC, and most mechanistic insight still comes from coronary and aortic beds rather than tibial arteries.^6^^,^^8^

The molecular drivers of tibial MAC in PAD remain poorly defined.^3^ Experimental studies implicate osteogenic reprogramming of vascular smooth muscle cells (VSMCs), inflammatory signaling, and metabolic stress responses in vascular calcification, but human data specifically focused on tibial arteries in PAD/CLTI is scarce.^6^ Diabetes remains highly prevalent in PAD and is strongly associated with both MAC and limb loss, raising the possibility that hyperglycemia influences calcification-related gene programs in tibial vessels.

High-throughput transcriptomic datasets offer an opportunity to systematically define PAD-associated gene programs and to identify candidate molecular targets for validation in human tissue. However, integrative approaches combining public datasets with validation in diseased human tibial arteries are limited. As human tibial arterial tissue is difficult to obtain, most available transcriptomic information comes from related lower-extremity gastrocnemius muscle biopsy, often in patients with claudication or animal samples. We therefore used public datasets and earlier literature to nominate a focused set of candidate genes with known or plausible roles in calcification-related pathways, matrix remodeling, inflammation, or metabolic stress, and then validated those candidates in human tibial arteries from patients with PAD as the primary disease-relevant tissue. To further probe the biologic context, we examined whether acute high-glucose exposure influenced expression in primary PAD-derived endothelial cells (ECs) and VSMCs derived from the same diseased vascular bed.

In this study, we used a stepwise, translational strategy to identify and validate candidate genes linked to calcification-relevant pathways in PAD-associated tibial arteries. We performed differential expression and pathway analyses across curated PAD/CLTI transcriptomic datasets, prioritized vascularly relevant candidate genes, and validated candidate expression in human tibial arteries. We then assessed whether high-glucose conditions altered candidate gene expression in primary ECs and VSMCs derived from PAD tibial arteries. We hypothesized that tibial MAC in PAD is associated with a dysregulated gene expression program that reflects chronic disease biology and may be driven by short-term hyperglycemia.

## Methods

### Study overview

We conducted a multistep study comprising: (1) integrative analysis of public PAD/CLTI transcriptomic datasets to identify differentially expressed genes (DEGs) and enriched pathways; (2) candidate gene prioritization based on vascular relevance, calcification biology, and cell-type mapping; (3) validation of candidate gene expression in human tibial arteries from patients with PAD and non-PAD patients; (4) gene expression in primary cells; and (5) in vitro assessment of hyperglycemia effects in primary PAD-derived tibial ECs and VSMCs.

### Public transcriptomic dataset selection and preprocessing

**Fig 1:**
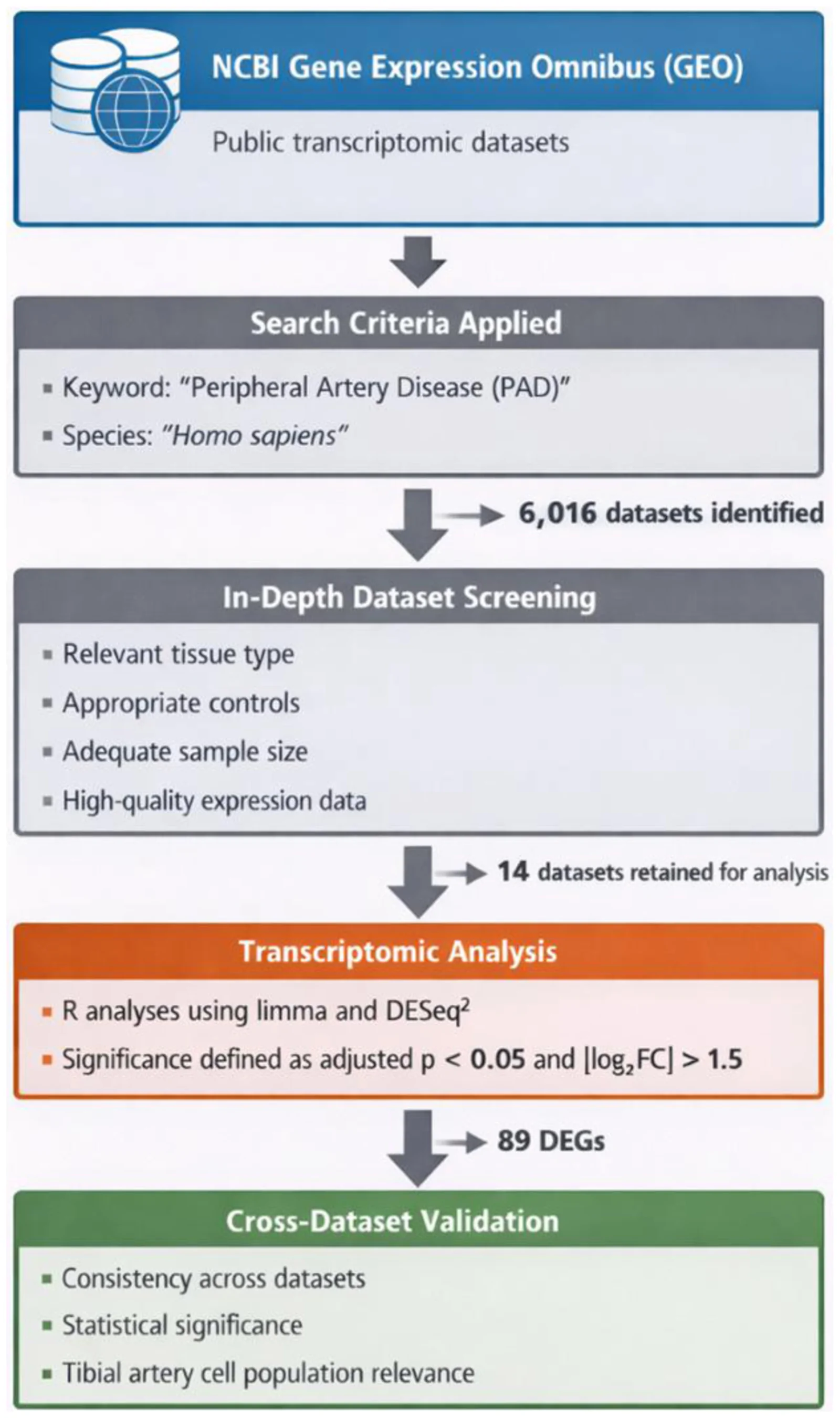
Systematic identification of peripheral artery disease (*PAD*)-related transcriptomic datasets from the National Center for Biotechnology Information (*NCBI*) gene expression omnibus (*GEO*) database, progressive screening and curation, differential gene expression (DEG) analysis, and validation of a refined set of PAD-associated DEGs.

Public gene expression datasets (GSE 27034, 37824, 42672, 49885, 84012, 113873, 120642, 197727, 227075, 248609, 266788, 275811, 275812, and 287300) were identified through a systematic search of the gene expression omnibus (GEO) using terms including “peripheral artery disease” and “limb ischemia,” restricted to *Homo sapiens*. Inclusion criteria were as follows: (1) human PAD/CLTI and non-PAD tissue samples; (2) adequate sample size for comparative analysis; (3) availability of raw or normalized expression data; and (4) sufficient clinical annotation allowing group assignment. In total, 14 datasets (n = 78 patients) met criteria (Fig 1); GSE120642 proved most informative and served as the principal dataset from which the candidate gene list was derived (Supplementary Fig 1, online only). Most included datasets did not independently yield candidate genes that met prioritization criteria; therefore, dataset-level DEG results and sample descriptions are provided in the *Supplementary Material*, whereas the main analysis emphasizes cross-dataset consistency and the most informative discovery dataset (Supplementary Table I, online only). Datasets derived from nonhuman species were excluded from the analysis; these are noted in Supplementary Table II (online only) for completeness.

### Differential expression and pathway analyses

Public transcriptomic datasets were analyzed to nominate candidate genes for targeted validation in human tibial arteries. Significance was defined as an adjusted *P* value < .05 using the Benjamini-Hochberg method and an absolute log2 fold-change >1.5. Genes with consistent directionality across datasets were considered PAD-associated.

Pathway and gene-set enrichment analyses were performed using established databases (eg, gene ontology, Kyoto Encyclopedia of Genes and Genomes, and curated vascular/calcification gene sets) to identify enriched biological processes and pathways among DEGs, with focus on inflammatory, osteogenic, and metabolic stress pathways.

### Candidate gene prioritization

From the set of PAD-associated DEGs, we applied a literature-guided prioritization framework rather than additional statistical filtering (Supplementary Table II, online only). Candidates were required to show consistent dysregulation in the discovery dataset and to have published evidence linking them to vascular remodeling, inflammation, lipid or metabolic stress, or extracellular matrix/calcification pathways, with endothelial and VSMC relevance annotated from previous experimental and transcriptomic studies (Table). Cell-type-specific relevance of candidate genes was inferred from previous experimental and transcriptomic studies rather than scRNA-seq within the present dataset. Single-cell and single-nucleus RNA-seq resources related to peripheral vascular disease were screened during dataset selection; however, available datasets were either nonhuman or did not yield genes meeting our differential expression criteria and were therefore not included in the integrative analysis (Supplementary Table I, online only). For each candidate gene, EC and VSMC relevance was assigned on the basis of published reports of expression and function in these cell types, as detailed in Table.

**Table:** Differentially expressed genes (DEGs) in peripheral artery disease (*PAD*) with cell-type specific relevance

Genes^a^	Expression	Primary function	EC relevance	VSMC relevance	Supporting literature/rationale
*ADAM12*	↑	ECM remodeling; metalloprotease activity; fibrosis	Moderate	High	PAD/vascular relevance; supports inclusion as a vascular remodeling candidate^9^
*ANGPTL4*	↑	Angiogenesis modulation; lipid metabolism; permeability	High	Moderate	Links ANGPTL4 to calcified vascular disease and vascular biology^10^
*CHI3L1*	↑	Inflammation; tissue remodeling; macrophage–EC crosstalk	High	Moderate	Directly supports CHI3L1 in arterial calcification biology^11^
*CYR61 (CCN1)*	↑	Cell adhesion; angiogenesis; mechanotransduction	High	High	Supports vascular remodeling and a calcification-related role^12^
*FDXR*	↑	Mitochondrial redox metabolism; oxidative stress response	Moderate	High	Metabolic/mitochondrial stress candidate with potential role in calcification^13^
*MYBPH*	↑	Cytoskeletal organization; contractile phenotype regulation	Low	High	Supports relevance to VSMC phenotype and remodeling^14^
*NNMT*	↑	Metabolic reprogramming; epigenetic regulation	Moderate	Moderate	Metabolic stress candidate with potential role in calcification^15^
*NPR3*	↑	Natriuretic peptide clearance; vascular tone regulation	Moderate	High	Vascular signaling candidate^16^
*PDK4*	↑	Mitochondrial metabolism; glucose-fatty acid switching	Moderate	High	Strong direct evidence for vascular calcification^17^^,^^18^
*PLA2G2A*	↑	Lipid signaling; inflammatory mediator generation	Moderate	High	Supports vascular inflammatory remodeling^19^
*PLTP*	↑	Lipid transport; HDL remodeling	High	Low-Moderate	Supports lipid/vascular disease relevance^20^
*RUNX1*	↑	Transcriptional regulation; inflammatory phenotypic switching	Moderate	High	Strong vascular transcriptional remodeling candidate with indirect link to calcification^21^^,^^22^
*SERPINA3*	↑	Protease inhibition; cellular energy tissue protection	Moderate	High	Supports inclusion in protease-inhibition/vascular remodeling pathways^23^
*FABP3*	↓	Fatty acid transport	Low	High	Strong PAD/metabolic relevance with indirect link to calcification^24^

*ANGPTL4*, Angiopoietin-like 4; *EC*, endothelial cell; *ECM*, extracellular matrix; *HDL*, high-density lipoprotein; *VSMC*, vascular smooth muscle cell.

Candidate prioritization was guided by literature-supported roles in vascular remodeling, calcification-associated pathways, inflammation, and metabolic stress, summarized in the final column. See references 10, 11, 12, 13, 14, 15, 16, 17, 18, 19, 20, 21, 22, 23, 24, 9 for supporting literature.

↑ = upregulated, ↓ = downregulated.

### Human tibial artery specimens

Tibial artery samples were obtained from patients undergoing below-the-knee amputation for PAD/CLTI and from non-PAD controls. All protocols were approved by the University of California San Diego Institutional Review Board (IRB No. 810512) with appropriate informed consent.

Arteries were excised from the amputated limb, carefully dissected free of surrounding nerve, vein, and soft tissue, and flushed with 10 mL of heparinized saline within 20 minutes of amputation. The proximal and distal 5-mm segments were reserved for histologic analysis, and the remaining vessel was processed for RNA and cell isolation. Tissue allocated for RNA analysis was snap-frozen in liquid nitrogen, and clinical variables, including diabetes status, were recorded.

### RNA isolation and quantitative PCR

Total RNA was extracted from frozen tibial artery segments using Direct-zol RNA MiniPrep Plus (Cat. No. R2072; Zymo Research) following the manufacturing recommended protocol. Complementary DNA was prepared from 1.0 μg total RNA by random priming using ProtoScript II First Strand cDNA Synthesis Kit (Cat. No. E6560S; New England Biolabs) following the manufacturing recommended for random primers protocol. Primers sequence for the SYBR green quantitative polymerase chain reaction (qPCR) of the 14 candidate genes were as follows: *ADAM12* (a disintegrin and metalloproteinase 12) forward: ACCGAGAGTTTCAGAGGCAA; reverse: CTGGTGAATGGGTCCTGACT. *ANGPTL4* forward: GGGTCTGGAGAAGGTGCATA; reverse: GTGGAGAAGGGTACGGAGAG. *CHI3L1* forward: TTCCCTCTCACCAATGCCAT; reverse: AGGGCTGAGCTCAAATCTGT. *CYR61* forward: ATGGTCCCAGTGCTCAAAGA; reverse: GGGCCGGTATTTCTTCACAC. *FDXR* forward: GGCCATGCGTTCATTCATCT; reverse: AGGACACAGGCTGCCTTATT. *MYBPH* (Myosin binding protein H) forward: ACACTGAAGAGTCACGGGAG; reverse: AAGGAAGGCAGGGAAAGGTT. *NNMT* forward: CCTAGACGGTGTGAAGGGAG; reverse: TGGACCCTTGACTCTGTTCC. *NPR3* forward: TGATGGTGTTCAGAGGGACC; reverse: TGCTGACACATCCTTGGAGT. *PDK4* (Pyruvate dehydrogenase kinase 4) forward: TTGGCTGGTTTTGGTTACGG; reverse: CACCAGTCATCAGCCTCAGA. *PLA2G2A* forward: ATGGGGAGAGGGAGTTGTTG; reverse: TCCCCTGAGCTGCATTTGTA. *PLTP* (Phospholipid transfer protein) forward: CTTCCTCCTCAACCAGCAGA; reverse: CCAGTTCCTCTCAGTCAGGG. *RUNX1* (Runt-related transcription factor 1) forward: TGTGATGGCTGGCAATGATG; reverse: TGATGGCTCTGTGGTAGGTG. *SERPINA3* forward: GCCTTTGCCACTGACTTTCA; reverse: TGCAAACTCATCATGGGCAC. *FABP3* (Fatty acid-binding protein 3) forward: GTGACACTGGATGGAGGGAA; reverse: GATACCACCCTGACCCACAA.

qPCR was performed with gene-specific primers and *GAPDH* as the reference gene. Reactions were run in triplicate, and relative expression was calculated using the $2^{-\Delta \Delta Ct}$ method, normalized to *GAPDH*, and expressed relative to the mean of the control group.^25^^,^^26^

### Primary EC and VSMC isolation

ECs were isolated by filling the arterial lumen with a freshly prepared enzyme mix consisting of Dispase (1.3 mg/mL; Cat. No. 17105041; Fisher Scientific) and Hyaluronidase (2.4 kU/mL; Cat. No. H3884; Sigma-Aldrich), followed by incubation at 37°C for 30 minutes. After digestion, the lumen was gently flushed, and the resulting cell suspension was collected in a 15 mL centrifuge tube, filtered through a 70 μm cell strainer, centrifuged at 300 *g* for 5 minutes, and the pellet resuspended in Endothelial Cell Growth Medium (Cat. No. 211-500; Cell applications, Inc) supplemented with 10% (vol./vol.) fetal bovine serum (FBS; Cat. No. F0926; Sigma-Aldrich) and 1% penicillin streptomycin (Pen Strep; Cat. No. 15140-122; Gibco). Cells were then seeded into culture plates, and after 24 hours, the medium was replaced to remove nonadherent cells. ECs were maintained at 37°C in a 5% CO_2_ atmosphere until further use.

Sequentially, after EC isolation the vessels were cut transversally, and the tunica media was dissected from the adventitia, minced, and incubated in the enzyme mix consisting of collagenase I (1.5 mg/mL; Cat. No. 17100017; Fisher Scientific), Dispase (1.6 mg/mL; Cat. No. 17105041; Fisher Scientific), and hyaluronidase (1 KU/mL; Cat. No. H3884; Sigma-Aldrich), followed by incubation at 37°C for 50 minutes, vortexing every 10 minutes. After digestion, the cell suspension was collected in a 15 mL centrifuge tube, filtered through a 70 μm cell strainer, centrifuged at 300 *g* for 5 minutes, and the pellet resuspended in Dulbecco's modified Eagle's medium (DMEM; Cat. No. 12430054; Gibco) supplemented with 10% (vol./vol.) FBS (Cat. No. F0926, Sigma-Aldrich) and 1× and 1% penicillin streptomycin (Pen Strep; Cat. No. 15140-122; Gibco) in humid air with 5% CO_2_ at 37°C.

EC identity was confirmed by cobblestone morphology and positive immunostaining for CD31 and VE-cadherin; VSMC identity was confirmed by spindle morphology and positive immunostaining for α-smooth muscle actin, consistent with previous characterization of cells isolated by sequential enzymatic digestion from human arteries.^9^ Successful high-glucose treatment was verified by defined media glucose concentrations; equimolar sucrose was used as an osmotic control to distinguish glucose-specific effects from osmotic stress.

### High-glucose vs osmotic control experiments

PAD-derived ECs and VSMCs were cultured for 7 days in either high-glucose media (25 mM glucose) or media supplemented with equimolar sucrose as an osmotic control. Media was refreshed every 2 to 3 days. RNA was collected at day 7, cDNA prepared, and qPCR performed for *ADAM12*, *PDK4*, and *RUNX1* as described above. Cell identity was confirmed using morphology and lineage-appropriate culture conditions, along with standard endothelial and smooth muscle surface markers during early passages.

### Statistical analysis

qPCR data are presented as means ± standard deviation. Group comparisons (PAD vs non-PAD; high-glucose vs osmotic control) were analyzed using Welch *t*-test to account for unequal variances. A *P* value < .05 was considered statistically significant. Given the exploratory nature and modest sample sizes, findings from cell culture experiments are interpreted as hypothesis generating.

## Results

### DEGs and pathway enrichment in PAD

Systematic screening of GEO identified 14 PAD/CLTI tissue datasets meeting the inclusion criteria. Differential expression analysis across datasets nominated 89 candidate genes enriched in pathways related to inflammation, innate immune signaling, extracellular matrix organization, osteogenic differentiation, mitochondrial metabolism, and oxidative stress (Fig 1).

Upregulated genes predominantly reflected: extracellular matrix remodeling, fibrosis, proinflammatory signaling, and lipid/energy metabolism. *FABP3* was uniquely downregulated, consistent with impaired fatty acid handling in PAD muscle (Table). During analysis, several datasets were excluded because they were nonhuman, whereas a small number of human datasets with very limited sample size (n < 5) were retained to preserve completeness of the search. These small datasets did not independently yield significant differentially expressed genes and therefore did not contribute to the final candidate list. Dataset-level DEG outputs and pathway analyses for the key discovery dataset are provided in the *Supplementary Material*, along with a summary of DEG counts across all included datasets.

### Prioritization of vascular and calcification-relevant genes

Of 89 DEGs, we prioritized 14 genes based on previous literature supporting roles in vascular remodeling, inflammatory signaling, lipid metabolism, extracellular matrix dynamics, and calcification-associated pathways (Table).^10^, ^11^, ^12^, ^13^, ^14^, ^15^, ^16^, ^17^, ^18^, ^19^, ^20^, ^21^, ^22^, ^23^, ^24^^,^^27^

### *ADAM12*, *PDK4*, and *RUNX1* are dysregulated in human PAD tibial arteries

**Fig 2:**
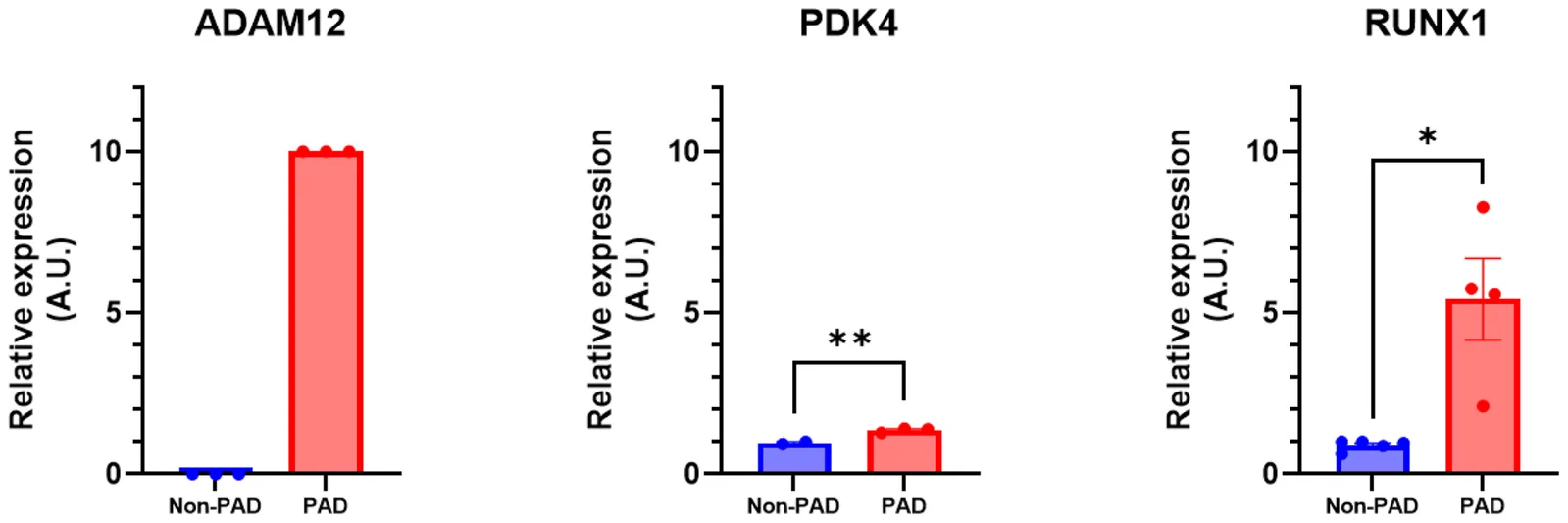
Relative gene expression of *ADAM12*, *PDK4*, and *RUNX1* in human tibial arteries from patients with peripheral artery disease (*PAD*; *red*) vs non-PAD (*blue*). Data are expressed as means ± SD. Statistical significance was determined by Welch *t*-test (∗*P* < .05, ∗∗*P* < .005). *PDK4* and *RUNX1* were significantly upregulated in PAD tibial arteries, whereas *ADAM12* showed a trend toward increased expression. *ADAM12*, A disintegrin and metalloproteinase 12; *AU*, arbitrary units; *PDK4*, pyruvate dehydrogenase kinase 4; *RUNX1*, runt-related transcription factor 1.

To validate these findings in disease-relevant tissue, gene expression was assessed in human tibial arteries from PAD and non-PAD patients. qPCR confirmed significant upregulation of PDK4 and *RUNX1* in PAD tibial arteries, with *ADAM12* showing a concordant but nonsignificant trend in the same direction (Fig 2). Functionally, these genes align with extracellular matrix remodeling (*ADAM12*), metabolic stress adaptation (*PDK4*), and inflammatory phenotypic switching and transcriptional control (*RUNX*) within ECs and VSMCs. MYBPH and PLTP, also demonstrated a PAD-restricted “on/off” expression pattern similar to *ADAM12*, but *ADAM12* was prioritized for detailed follow-up because of its higher predicted relevance in both ECs and VSMCs. In contrast, *FABP3* exhibited higher expression in PAD than non-PAD arteries, opposite to the downregulation predicted from the discovery dataset, and was therefore not pursued as a lead candidate (Supplementary Fig 2, online only).

### Hyperglycemia does not further increase candidate gene expression in PAD, ECs, and VSMCs

Given the strong epidemiologic association between diabetes, MAC, and limb loss, we next tested the impact of high-glucose conditions on further augmenting the expression of *ADAM12, PDK4*, and *RUNX1* in PAD vascular cells.

**Fig 3:**
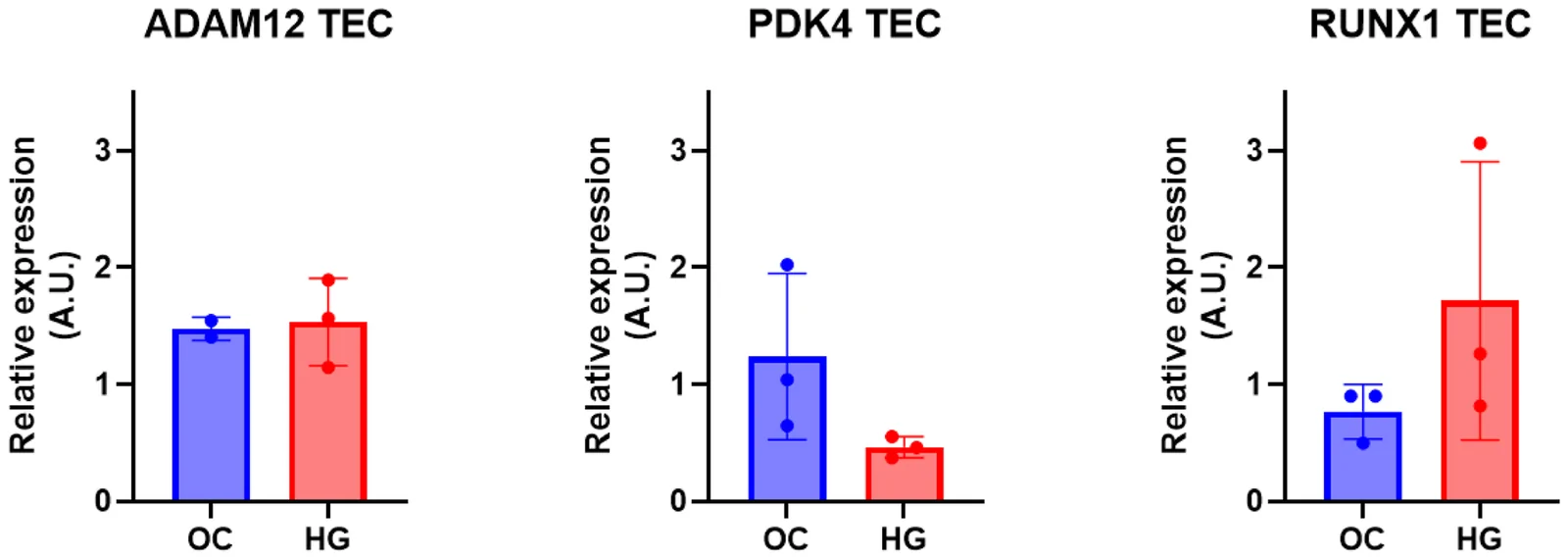
Relative gene expression of *ADAM12*, *PDK4*, and *RUNX1* in primary endothelial cells (*ECs*) isolated from peripheral artery disease (PAD) tibial arteries cultured for 7 days under osmotic control (*OC*) vs high glucose (*HG*) conditions. Data are expressed as means ± SD. No significant differences were observed between conditions (Welch *t-*test, *P* > .05). HG exposure did not further augment expression of these PAD-associated procalcific genes in ECs. *ADAM12*, A disintegrin and metalloproteinase 12; *AU*, arbitrary units; *PDK4*, pyruvate dehydrogenase kinase 4; *RUNX1*, runt-related transcription factor 1.

**Fig 4:**
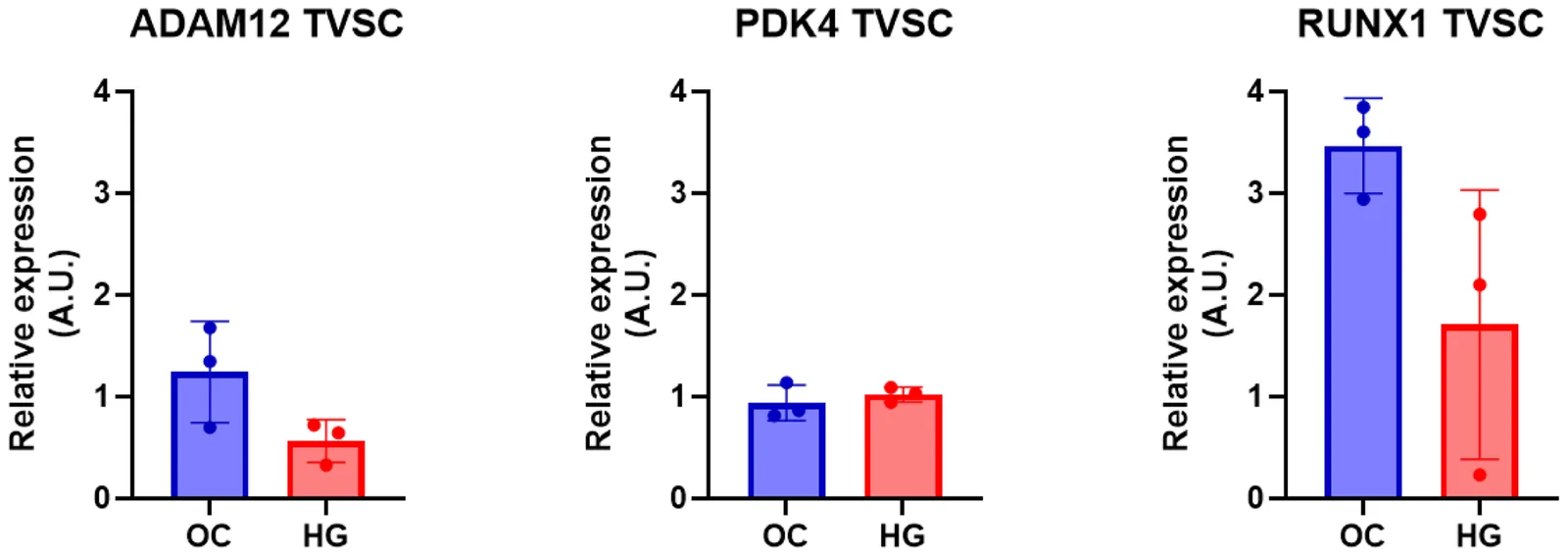
Relative gene expression of *ADAM12*, *PDK4*, and *RUNX1* in primary vascular smooth muscle cells (*VSMCs*) isolated from peripheral artery disease (PAD) tibial arteries cultured for 7 days under osmotic control (*OC*) vs high glucose (*HG*) conditions. Data are expressed as means ± SD. No significant differences were observed between conditions (Welch *t*-test, *P* > .05). HG exposure did not further augment expression of these PAD-associated procalcific genes in VSMCs, the primary site of MAC. *ADAM12*, A disintegrin and metalloproteinase 12; *AU*, arbitrary units; *MAC*, medial arterial calcification; *PDK4*, pyruvate dehydrogenase kinase 4; *RUNX1*, runt-related transcription factor 1.

Across replicate experiments, acute high-glucose exposure did not meaningfully alter expression of *ADAM12, PDK4,* or *RUNX1* in PAD-derived ECs or VSMCs compared with osmotic controls (Figs 3 and 4). These data suggest that acute high-glucose exposure, over a period of 7 days, does not further drive the gene program defined by *ADAM12, PDK4*, and *RUNX1* PAD-derived vascular cells.

## Discussion

This study demonstrates how literature-guided candidates from public transcriptomic datasets can be validated in human tibial arteries and tested in primary PAD-derived vascular cells to identify *ADAM12, PDK4*, and *RUNX1* as PAD-associated genes enriched in calcified tibial vessels, rather than as definitively established calcification drivers. In tibial arteries, *PDK4* and *RUNX1* were significantly upregulated in PAD, whereas *ADAM12* exhibited a similar PAD-enriched expression pattern that did not reach statistical significance in this modest cohort; we therefore treat *ADAM12* as a prioritized candidate rather than a fully validated marker. These genes converge on extracellular matrix remodeling, metabolic reprogramming, and inflammatory transcriptional control, defining coordinated programs that may contribute to MAC in PAD/CLTI but will require future functional experiments to establish causal roles.

### *ADAM12*, *PDK4*, and *RUNX1* as calcification-relevant tibial targets

*ADAM12*, a metalloprotease implicated in extracellular matrix turnover and fibrosis, was upregulated in PAD tibial arteries, consistent with enhanced matrix remodeling and increased medial stiffness conditions that may favor calcification.^10^^,^^28^^,^^29^ *PDK4*, a regulator of mitochondrial metabolism, shifts substrate utilization toward fatty acid oxidation and reflects metabolic stress, a known contributor to vascular calcification and stiffness.^19^^,^^30^ *RUNX1*, best known for roles in hematopoiesis and leukemogenesis, also participates in inflammatory signaling and phenotypic switching in vascular cells.^31^ Enhanced *RUNX1* expression in PAD tibial arteries may drive transcriptional programs that favor proinflammatory, osteogenic, or proliferative states in VSMCs and ECs. Together, these genes form a plausible module in which extracellular matrix remodeling, metabolic reprogramming, and inflammatory transcriptional control converge to influence tibial arterial remodeling and MAC.

### Hyperglycemia as a modifier vs driver

Although diabetes is strongly associated with MAC and limb loss, the mechanisms remain incompletely understood.^13^^,^^32^ Our data suggest that an acute high-glucose exposure is not a dominant driver of *ADAM12, PDK4,* or *RUNX1* expression in PAD-derived ECs and VSMCs over 7 days, and are therefore more consistent with these genes being influenced by the broader chronic PAD-associated vascular milieu rather than by short-term hyperglycemia alone. This pattern suggests that dysregulation of these genes in PAD tibial arteries is likely shaped by the cumulative impact of ischemia, inflammation, long-standing metabolic alterations, and other coexisting risk factors, rather than attributable solely to acute glycemic status. These results do not exclude an important role for diabetes in modulating calcification through mechanisms such as advanced glycation endproducts, oxidative stress, or alterations in mineral metabolism, but they underscore the need to consider PAD and its associated chronic stimuli as key contributors to calcification-relevant signaling in tibial arteries.

### Therapeutic implications

*ADAM12, PDK4*, and *RUNX1* represent actionable targets within key pathways linked to calcification. Each occupies a nodal position in remodeling, metabolic, or inflammatory pathways, and pharmacologic modulators or inhibitory strategies such as small molecule inhibitors, RNA interference, or gene-editing approaches are conceptually feasible. Moreover, tibial arteries are accessible to local delivery modalities, including drug-coated balloons, stents, or perivascular delivery platforms, which could minimize systemic exposure.

Targeting these nodes could, in principle, slow or reverse the progression of MAC, improve vessel compliance, and enhance the technical success and durability of infrapopliteal revascularization. Such strategies would complement rather than replace standard PAD therapies and might be especially beneficial for patients with heavily calcified tibial disease.

### Limitations

This study has several limitations. First, the initial discovery phase relied on public transcriptomic datasets from lower-extremity muscle rather than tibial arteries. This tissue mismatch is a major limitation, as muscle-derived expression signatures may not fully reflect vessel-specific processes such as medial arterial calcification. Although using muscle enabled an adequate sample size and clinical annotation, it necessitates cautious interpretation of muscle-based candidate nomination and underscores the need for future discovery work directly in diseased tibial arteries. Second, our tibial artery validation cohort was modest in size and lacked complete data on diabetes status, glycemic control (eg, glycated hemoglobin), and calcification severity, limiting our ability to perform adequately powered stratified analyses or determine whether gene expression differences are driven by PAD itself, coexisting diabetes, or calcific burden. Third, the in vitro high-glucose experiments used a 7-day exposure paradigm and therefore do not exclude the possibility that longer-term or chronic diabetic conditions could modulate *ADAM12, PDK4*, or *RUNX1* expression through mechanisms not captured in this acute setting. As the high-glucose experiments were performed only in PAD-derived ECs and VSMCs, we could not determine whether *ADAM12, PDK4*, or *RUNX1* remain glucose-responsive in healthy non-PAD vascular cells, an important question for future studies. In addition, we did not reassess lineage markers or glycolytic gene expression during the high-glucose experiments, so our conclusions about hyperglycemic responsiveness are based on defined media conditions rather than molecular verification of glycolytic pathway activation. Cell-type specificity of candidate genes was inferred from published experimental and transcriptomic studies rather than confirmed using human tibial artery single-cell RNA sequencing datasets, which remain limited in availability. Future studies incorporating single-cell approaches will be important to further refine cellular localization of these candidate pathways. Finally, our work focused on gene expression rather than functional outcomes; we did not directly test whether manipulating *ADAM12, PDK4*, or *RUNX1* alters osteogenic differentiation, calcium deposition, or VSMC phenotype, and thus cannot infer a causal role in medial calcification.

### Future directions

Future studies should expand validation to larger, prospectively collected tibial artery cohorts. Functional experiments using loss- and gain-of-function approaches in human ECs and VSMCs, as well as in vivo models of tibial MAC, are needed to define causality and therapeutic potential. Integration with histopathologic analysis, advanced imaging, proteomics, and extracellular vesicle profiling may also identify noninvasive biomarkers of *ADAM12*, *PDK4*, and *RUNX1* activity and guide patient selection for targeted interventions.

## Conclusions

Integrative analysis of PAD/CLTI transcriptomic datasets with validation in human tibial arteries, identifies *ADAM12*, *PDK4*, and *RUNX1* as novel PAD-associated, calcification-relevant targets. These genes link extracellular matrix remodeling, metabolic stress, and inflammatory transcriptional control to MAC, and are not further upregulated by short-term hyperglycemia in PAD vascular cells. Together, *ADAM12*, *PDK4*, and *RUNX1* represent promising targets to limit tibial MAC and improve limb-salvage in PAD and CLTI.

## Author Contributions

Conception and design: SA, JR, BE, AG

Analysis and interpretation: SA, JR, BE, AG

Data collection: SA, JR, AG

Writing the article: SA, JR

Critical revision of the article: SA, JR, BE, AG

Final approval of the article: SA, JR, BE, AG

Statistical analysis: SA, JR

Obtained funding: BE, AG

Overall responsibility: AG

S.A. and J.R. contributed equally to this work and share co-first authorship.

## Funding

This work was supported by the UCSD Department of Surgery Faculty Research grant.

## Disclosures

None.

## Appendix (online only)

Supplementary Table I

Supplementary Table II

## References

[1] Deng L., Du C., Liu L. (2025). Forecasting the global burden of peripheral artery disease from 2021 to 2050: a population-based study. Research (Wash D C).

[2] Fowkes F.G.R., Rudan D., Rudan I. (2013). Comparison of global estimates of prevalence and risk factors for peripheral artery disease in 2000 and 2010: a systematic review and analysis. Lancet.

[3] Alabi O., Rokosh R., Zheng X. (2026). Progression to chronic limb-threatening ischemia after index revascularization for claudication. JAMA Surg.

[4] Zettervall S.L., Marshall A.P., Fleser P., Guzman R.J. (2018). Association of arterial calcification with chronic limb ischemia in patients with peripheral artery disease. J Vasc Surg.

[5] Losurdo F., Casini A., Clerici G., Ferraresi R. (2021). Medial artery calcification: the silent killer of the leg. J Am Coll Cardiol.

[6] Kim T.I., Guzman R.J. (2023). Medial artery calcification in peripheral artery disease. Front Cardiovasc Med.

[7] Van den Branden A., Verhulst A., D'Haese P.C., Opdebeeck B. (2022). New therapeutics targeting arterial media calcification: friend or foe for bone mineralization?. Metabolites.

[8] Salle L., Magne J., Kenne Malaha A. (2023). Ultrasound-detected tibial artery calcification as a marker of cardiovascular and lower-limb risk in asymptomatic patients with type-2 diabetes. Vasc Med.

[9] Moss S.C., Bates M., Parrino P.E., Woods T.C. (2007). Isolation of endothelial cells and vascular smooth muscle cells from internal mammary artery tissue. Ochsner J.

[10] Dokun A.O., Chen L., Okutsu M. (2015). ADAM12: a genetic modifier of preclinical peripheral arterial disease. Am J Physiol Heart Circ Physiol.

[11] Katano H., Yamada K. (2014). Upregulation of ANGPTL4 messenger RNA and protein in severely calcified carotid plaques. J Stroke Cerebrovasc Dis.

[12] Zhu Y., Chen J.C., Zhang J.L., Wang F.F., Liu R.P. (2025). A new mechanism of arterial calcification in diabetes: interaction between H3K18 lactylation and CHI3L1. Clin Sci (Lond).

[13] Grzeszkiewicz T.M., Lindner V., Chen N., Lam S.C., Lau L.F. (2002). The angiogenic factor cysteine-rich 61 (CYR61, CCN1) supports vascular smooth muscle cell adhesion and stimulates chemotaxis through integrin alpha(6)beta(1) and cell surface heparan sulfate proteoglycans. Endocrinology.

[14] Lu Y., Wu Y., Yang C. (2025). Ferredoxins: master regulators in mitochondrial redox homeostasis and programmed cell death. Redox Biol.

[15] Zhu T., He Y., Yang J., Fu W., Xu X., Si Y. (2017). MYBPH inhibits vascular smooth muscle cell migration and attenuates neointimal hyperplasia in a rat carotid balloon-injury model. Exp Cell Res.

[16] Campagna R., Mateuszuk Ł., Wojnar-Lason K. (2021). Nicotinamide N-methyltransferase in endothelium protects against oxidant stress-induced endothelial injury. Biochim Biophys Acta Mol Cell Res.

[17] Chinetti G., Neels J.G. (2021). Roles of nuclear receptors in vascular calcification. Int J Mol Sci.

[18] Lee S.J., Jeong J.Y., Oh C.J. (2015). Pyruvate dehydrogenase kinase 4 promotes vascular calcification via SMAD1/5/8 phosphorylation. Sci Rep.

[19] Ma W.Q., Sun X.J., Zhu Y., Liu N.F. (2020). PDK4 promotes vascular calcification by interfering with autophagic activity and metabolic reprogramming. Cell Death Dis.

[20] Wang X., Li S., Liu C. (2024). High expression of PLA2G2A in fibroblasts plays a crucial role in the early progression of carotid atherosclerosis. J Transl Med.

[21] Schlitt A., Bickel C., Thumma P. (2003). High plasma phospholipid transfer protein levels as a risk factor for coronary artery disease. Arterioscler Thromb Vasc Biol.

[22] Chen M.J., Yokomizo T., Zeigler B.M., Dzierzak E., Speck N.A. (2009). Runx1 is required for the endothelial to haematopoietic cell transition but not thereafter. Nature.

[23] Yzaguirre A.D., Howell E.D., Li Y., Liu Z., Speck N.A. (2018). Runx1 is sufficient for blood cell formation from non-hemogenic endothelial cells in vivo only during early embryogenesis. Development.

[24] Towler D.A. (2015). Arteriosclerotic calcification: a serpi(n)ginous path to cardiovascular health?. Circ Res.

[25] Livak K.J., Schmittgen T.D. (2001). Analysis of relative gene expression data using real-time quantitative PCR and the 2(-Delta Delta C(T)) Method. Methods.

[26] Schmittgen T.D., Livak K.J. (2008). Analyzing real-time PCR data by the comparative C(T) method. Nat Protoc.

[27] Zamzam A., Syed M.H., Rotstein O.D. (2022). Validating fatty acid binding protein 3 as a diagnostic and prognostic biomarker for peripheral arterial disease: a three-year prospective follow-up study. EClinicalMedicine.

[28] Lamin V., Verry J., Dokun O.S. (2022). microRNA-29a regulates ADAM12 through direct interaction with ADAM12 mRNA and modulates postischemic perfusion recovery. J Am Heart Assoc.

[29] Wang Z., Zhao X., Zhao G. (2023). PRDM16 deficiency in vascular smooth muscle cells aggravates abdominal aortic aneurysm. JCI Insight.

[30] Ma W., Sun X., Zhu Y., Liu Y. (2020). PDK4 promotes vascular calcification by interfering with autophagic degradation. Cell Death Dis.

[31] Cao G., Xuan X., Hu J., Zhang R., Jin H., Dong H. (2022). How vascular smooth muscle cell phenotype switching contributes to vascular disease. Cell Commun Signal.

[32] Chistiakov D.A., Sobenin I.A., Orekhov A.N., Bobryshev Y.V. (2014). Mechanisms of medial arterial calcification in diabetes. Curr Pharm Des.

