# Supplementary material for: Integrative transcriptomics and human tibial artery validation identify ADAM12, PDK4, and RUNX1 as peripheral artery disease-associated genes enriched in calcified tibial arteries
Source: JVS Vasc Sci. 2026 Jul 27;7:100434. doi: 10.1016/j.jvssci.2026.100434 (PMC13571584; doi:10.1016/j.jvssci.2026.100434)
Supplement: Supplementary file 1 [file mmc2.docx]

| **GEO Accession** | **First author** | **Year** | **Tissue / Sample Type** | **Platform** | **Sample Size** | **DEGs** | **Contribution to Analysis** |
| --- | --- | --- | --- | --- | --- | --- | --- |
| GSE37824 | Puig | 2012 | Vessel plaque | Microarray | 121 | 0 | Supporting dataset |
| GSE197727 | Ring | 2022 | Myotubes | RNA-seq | 3 | 4 | Supporting dataset |
| GSE113873 | Parmer | 2021 | Skeletal muscle | Microarray | 52 | 0 | Supporting dataset |
| GSE120642 | Ryan | 2018 | Skeletal muscle | RNA-seq | 51 | 89 | Key discovery dataset |
| GSE49885 | Dangwal | 2015 | Plasma | Microarray | 37 | 9 | Supporting dataset |
| GSE42672 | Li | 2012 | Fibroblasts | Microarray | 4 | 0 | Supporting dataset |
| GSE27034 | Masud | 2012 | Monocytes | Microarray | 37 | 0 | Supporting dataset |
| GSE84012 | Espitia | 2018 | Smooth muscle | Microarray | 4 | 0 | Supporting dataset |
| GSE275812 | Zhu | 2024 | Mice myotubes | RNA-seq | N/A | N/A | Excluded for non-human |
| GSE275811 | Zhu | 2024 | Mice myotubes | RNA-seq | N/A | N/A | Excluded for non-human |
| GSE248609 | Qian | 2023 | Artery | RNA-seq | 11 | 0 | Supporting dataset |
| GSE287300 | Turiel | 2025 | Skeletal muscle | RNA-seq | 8 | 0 | Supporting dataset |
| GSE266788 | Cartland | 2024 | Skeletal muscle | RNA-seq | 1 | 0 | Supporting dataset |
| GSE227075 | Southerland | 2023 | Mice skeletal muscle | RNA-seq | N/A | N/A | Excluded for non-human |

Table S1. Summary of GEO datasets included in the transcriptomic analysis, showing accession number, publication details, tissue type, platform, sample size, and each dataset’s contribution to candidate-gene prioritization. Non-human datasets were excluded; a few small human datasets (n < 5) were retained for completeness but did not yield significant DEGs.
