# Supplementary material for: Integrative transcriptomics and human tibial artery validation identify ADAM12, PDK4, and RUNX1 as peripheral artery disease-associated genes enriched in calcified tibial arteries
Source: JVS Vasc Sci. 2026 Jul 27;7:100434. doi: 10.1016/j.jvssci.2026.100434 (PMC13571584; doi:10.1016/j.jvssci.2026.100434)
Supplement: Supplementary file 2 [file mmc3.docx]

| **Gene** | **Name** | **Description** |
| --- | --- | --- |
| ADAM12 | ADAM metallopeptidase domain 12 | Metalloprotease involved in adhesion, migration, and tissue remodeling; roles in cancer, fibrosis, and vascular biology |
| ANGPTL4 | Angiopoietin-like 4 | Regulates angiogenesis, lipid metabolism, and vascular permeability; induced by hypoxia and fasting |
| ARHGAP36 | Rho GTPase activating protein 36 | Atypical RhoGAP modulating Hedgehog signaling and neuronal growth |
| BBC3 | BCL2 binding component 3 | Pro-apoptotic BH3-only protein, mediator of p53-dependent apoptosis |
| C4A | Complement component 4A | Classical complement pathway component; genetic variants linked to autoimmunity |
| C7 | Complement component 7 | Part of terminal complement complex (MAC) mediating cell lysis |
| CD68 | CD68 molecule | Macrophage glycoprotein; widely used monocyte/macrophage lineage marker |
| CDKN1A | Cyclin dependent kinase inhibitor 1A | CDK inhibitor; enforces cell cycle arrest in response to DNA damage |
| CHI3L1 | Chitinase 3 like 1 | Inflammation-associated glycoprotein; biomarker in arthritis, cancer, atherosclerosis |
| CHI3L2 | Chitinase 3 like 2 | Less characterized chitinase-like protein; possible role in tissue remodeling |
| CHRNA1 | Cholinergic receptor nicotinic alpha 1 subunit | Muscle-type nicotinic acetylcholine receptor subunit; mutations cause congenital myasthenic syndrome |
| COL19A1 | Collagen type XIX alpha 1 chain | Minor basement membrane collagen; mutations linked to myopathy |
| CP | Ceruloplasmin | Copper-binding ferroxidase essential for iron homeostasis; deficiency causes aceruloplasminemia |
| CPXM1 | Carboxypeptidase X M14 family member 1 | Putative metalloprotease; function unclear |
| CYR61 | Cysteine rich angiogenic inducer 61 | ECM-associated protein promoting angiogenesis, adhesion, wound healing |
| EFCAB7 | EF-hand calcium binding domain 7 | Calcium-binding protein; poorly characterized |
| EGR1 | Early growth response 1 | Immediate early transcription factor regulating proliferation, inflammation, vascular remodeling |
| EGR2 | Early growth response 2 | Transcription factor critical for myelination; mutations cause Charcot–Marie–Tooth disease |
| ERBB3 | Erb-b2 receptor tyrosine kinase 3 | EGF receptor family member; activates PI3K/AKT signaling, implicated in cancer |
| F13A1 | Coagulation factor XIII A chain | Enzyme crosslinking fibrin clots; deficiency causes bleeding disorder |
| FASN | Fatty acid synthase | Enzyme synthesizing fatty acids; overexpressed in cancers and metabolic diseases |
| FCGBP | Fc fragment of IgG binding protein | Mucin-associated protein in epithelial secretions; contributes to mucosal defense |
| FDXR | Ferredoxin reductase | Mitochondrial enzyme linking oxidative metabolism to apoptosis; p53-regulated |
| FOS | Fos proto-oncogene, AP‑1 transcription factor subunit | Immediate early transcription factor; regulates growth, stress, and inflammation |
| FOSB | FosB proto-oncogene, AP‑1 transcription factor subunit | AP‑1 transcription factor; roles in neuronal plasticity and stress responses |
| FST | Follistatin | Glycoprotein that binds activins/myostatin; regulates muscle growth and fibrosis |
| GADD45A | Growth arrest and DNA damage inducible alpha | Stress-response protein; regulates DNA repair, apoptosis, and cell cycle |
| GDNF | Glial cell derived neurotrophic factor | Supports neuronal survival and kidney development |
| HP | Haptoglobin | Binds free hemoglobin; antioxidant and anti-inflammatory; variants linked to CVD |
| HSD52 | HSD52 long noncoding RNA (LOC729467) | Long noncoding RNA; poorly characterized |
| MIR503HG | MIR503 host gene | lncRNA hosting miR‑503; roles in cell cycle regulation and cancer |
| MYBPH | Myosin binding protein H | Myofibrillar protein involved in sarcomere structure |
| NGFR | Nerve growth factor receptor | Receptor for neurotrophins; regulates neuronal survival, apoptosis, inflammation |
| NNMT | Nicotinamide N-methyltransferase | Enzyme in nicotinamide metabolism; implicated in cancer and energy balance |
| PDK4 | Pyruvate dehydrogenase kinase 4 | Inhibits pyruvate dehydrogenase, shifting metabolism to fatty acid oxidation |
| PLA2G2A | Phospholipase A2 group IIA | Secreted phospholipase with proinflammatory and antimicrobial activity; linked to atherosclerosis |
| PLTP | Phospholipid transfer protein | Transfers phospholipids between lipoproteins; regulates HDL metabolism and CVD risk |
| PNPLA3 | Patatin-like phospholipase domain containing 3 | Lipid remodeling enzyme; major NAFLD risk gene |
| PRUNE2 | Prune homolog 2 with BCH domain | Putative tumor suppressor; roles in neuronal differentiation |
| PTPRF | Protein tyrosine phosphatase receptor type F | Regulates adhesion and axon guidance; implicated in cancer |
| RIN1 | Ras and Rab interactor 1 | RAS effector regulating receptor endocytosis and signaling |
| RUNX1 | Runt-related transcription factor 1 | Master regulator of hematopoiesis; mutations cause leukemia |
| SAA1 | Serum amyloid A1 | Acute phase protein; inflammatory marker; can form amyloid deposits |
| SAA2 | Serum amyloid A2 | Acute phase protein similar to SAA1; inflammation marker |
| SCN5A | Sodium voltage-gated channel alpha subunit 5 | Cardiac sodium channel; mutations cause arrhythmias (e.g., Brugada syndrome) |
| SERPINA3 | Serpin family A member 3 | Protease inhibitor; upregulated in inflammation and neurodegeneration |
| SFRP2 | Secreted frizzled related protein 2 | Wnt pathway modulator; roles in angiogenesis and fibrosis |
| SLPI | Secretory leukocyte peptidase inhibitor | Antimicrobial and antiprotease; protects mucosal surfaces |
| SPON1 | Spondin 1 | ECM protein involved in axon guidance and vascular development |
| SRPX | Sushi repeat containing protein X‑linked | Extracellular protein; implicated in angiogenesis and synapse formation |
| METTL21EP | Methyltransferase like 21E pseudogene | Non-functional pseudogene |
| TRNQ | tRNA for glutamine | Mitochondrial tRNA for glutamine |
| COQ10A | Coenzyme Q10A | Involved in coenzyme Q10 biosynthesis; important for mitochondrial respiration |
| FABP3 | Fatty acid binding protein 3 | Heart-type fatty acid transporter; cardiac injury biomarker and lipid chaperone |
| PLEKHN1 | Pleckstrin homology domain containing N1 | Protein of unclear function; may regulate signaling via PH domain |
| CKMT2 | Creatine kinase mitochondrial 2 | Mitochondrial creatine kinase; energy buffering in muscle |
| ATP13A5 | ATPase 13A5 | P‑type ATPase; poorly characterized |
| BDH1 | 3‑hydroxybutyrate dehydrogenase 1 | Mitochondrial enzyme in ketone body metabolism |
| FAM151A | Family with sequence similarity 151 member A | Poorly characterized gene of unknown function |
| TRNM | tRNA for methionine | Mitochondrial tRNA for methionine |
| TRNV | tRNA for valine | Mitochondrial tRNA for valine |
| CYP1A1 | Cytochrome P450 family 1 subfamily A member 1 | Enzyme metabolizing xenobiotics and polycyclic aromatic hydrocarbons |
| RPS4Y1 | Ribosomal protein S4 Y-linked 1 | Small ribosomal subunit protein; Y‑linked isoform |
| EIF1AY | Eukaryotic translation initiation factor 1A Y-linked | Translation initiation factor subunit; Y‑linked variant |
| HBA2 | Hemoglobin subunit alpha 2 | Alpha-globin subunit; mutations cause alpha-thalassemia |
| HBA1 | Hemoglobin subunit alpha 1 | Alpha-globin subunit; nearly identical to HBA2; mutations cause alpha-thalassemia |
| KDM5D | Lysine demethylase 5D | Histone demethylase; Y‑linked; role in spermatogenesis |
| HBB | Hemoglobin subunit beta | Beta-globin subunit; mutations cause beta-thalassemia and sickle cell disease |
| VMO1 | Vitelline membrane outer layer 1 homolog | Extracellular protein of unclear function; vitelline membrane–related |
| PITX1 | Paired like homeodomain 1 | Transcription factor critical for hindlimb development; mutations cause Liebenberg syndrome |
| USP9Y | Ubiquitin specific peptidase 9 Y-linked | Deubiquitinating enzyme; Y‑linked; implicated in spermatogenesis |
| IGFN1 | Immunoglobulin like and fibronectin type III domain containing 1 | Large sarcomeric protein; skeletal muscle-specific |
| ANKRD1 | Ankyrin repeat domain 1 | Muscle stress-response protein; regulates hypertrophy and sarcomere signaling |
| KCNE1L | Potassium voltage-gated channel subfamily E regulatory subunit 1 like | Potassium channel regulator; influences cardiac repolarization |
| UTY | Ubiquitously transcribed tetratricopeptide repeat gene Y-linked | Y‑linked TPR gene; histone demethylase homolog |
| PF4 | Platelet factor 4 | Platelet chemokine; promotes platelet aggregation and leukocyte recruitment |
| PVALB | Parvalbumin | Calcium-binding protein in fast-twitch muscle and interneurons |
| NPR3 | Natriuretic peptide receptor 3 | Clearence receptor for natriuretic peptides; regulates vascular tone and cardiac remodeling |
| PPBP | Pro‑platelet basic protein | Platelet-derived chemokine (CXCL7); neutrophil recruitment, angiogenesis, wound healing |
| TXLNGY | Taxilin gamma Y-linked | Y‑linked protein involved in vesicle trafficking; poorly characterized |
| S100A8 | S100 calcium binding protein A8 | Neutrophil protein; mediator of inflammation and oxidative stress |
| XIST | X inactive specific transcript | lncRNA essential for X‑chromosome inactivation |
| CRYM | Crystallin mu | Binds thyroid hormone and NADPH; roles in metabolism and hearing |
| PRG4 | Proteoglycan 4 | Lubricating glycoprotein in synovial fluid; protects cartilage surfaces |
| PCOLCE2 | Procollagen C-endopeptidase enhancer 2 | Enhances collagen processing by BMP‑1; important for ECM maturation |
| SIM2 | SIM bHLH transcription factor 2 | Transcription factor in CNS and craniofacial development; part of Down syndrome critical region |

**Table S2. Differentially expressed genes (DEGs) identified in the discovery dataset GSE120642.** The table lists all DEGs retained after applying the predefined thresholds (adjusted p < 0.05 and |log2 fold‑change| > 1.5), including official gene symbol, full gene name, and a brief functional description based on curated gene annotations and prior literature.
